# Chronic Apical and Nonapical Right Ventricular Pacing in Patients with High-Grade Atrioventricular Block: Results of the Right Pace Study

**DOI:** 10.1155/2018/1404659

**Published:** 2018-05-22

**Authors:** Carmine Muto, Valeria Calvi, Giovanni Luca Botto, Domenico Pecora, Daniele Porcelli, Alessandro Costa, Gianfranco Ciaramitaro, Riccardo Airò Farulla, Anna Rago, Raimondo Calvanese, Marco Tullio Baratto, Albino Reggiani, Massimo Giammaria, Santina Patané, Monica Campari, Sergio Valsecchi, Giampiero Maglia

**Affiliations:** ^1^Ospedale S. Maria della Pietà, Nola, Italy; ^2^A.O.U.P. “Vittorio Emanuele”, Ospedale Ferrarotto, Catania, Italy; ^3^Ospedale Sant'Anna, San Fermo della Battaglia, Italy; ^4^Fondazione Poliambulanza, Brescia, Italy; ^5^Ospedale San Pietro, Roma, Italy; ^6^Ospedale Sacro Cuore Don Calabria, Negrar, Italy; ^7^A.O.U.P. “Paolo Giaccone”, Università degli Studi di Palermo, Palermo, Italy; ^8^Fondazione Istituto G. Giglio, Cefalù, Italy; ^9^Seconda Università degli Studi di Napoli, Ospedale Monaldi, Napoli, Italy; ^10^Ospedale Santa Maria di Loreto Mare, Napoli, Italy; ^11^Ospedale Versilia, Lido di Camaiore, Italy; ^12^Ospedale Carlo Poma, Mantua, Italy; ^13^Ospedale Maria Vittoria, Torino, Italy; ^14^Ospedali Riuniti Papardo, Messina, Italy; ^15^Boston Scientific Italia, Milano, Italy; ^16^Ospedale Pugliese-Ciaccio, Catanzaro, Italy

## Abstract

**Objective:**

The aim of the study was to compare the two approaches to chronic right ventricular pacing currently adopted in clinical practice: right ventricular apical (RVA) and non-RVA pacing.

**Background:**

Chronic RVA pacing is associated with an increased risk of atrial fibrillation, morbidity, and even mortality. Non-RVA pacing may yield more physiologic ventricular activation and provide potential long-term benefits and has recently been adopted as standard procedure at many implanting centers.

**Methods:**

The Right Pace study was a multicenter, prospective, single-blind, nonrandomized trial involving 437 patients indicated for dual-chamber pacemaker implantation with a high percentage of RV pacing.

**Results:**

RV lead-tip target location was the apex or the interventricular septum. RVA (274) and non-RVA patients (163) did not differ in baseline characteristics. During a median follow-up of 19 months (25th–75th percentiles, 13–25), 17 patients died. The rates of the primary outcome of death due to any cause or hospitalization for heart failure were comparable between the groups (log-rank test, *p* = 0.609), as were the rates of the composite of death due to any cause, hospitalization for heart failure, or an increase in left ventricular end-systolic volume ≥ 15% as compared with the baseline evaluation (secondary outcome, *p* = 0.703). After central adjudication of X-rays, comparison between adjudicated RVA (239 patients) and non-RVA (170 patients) confirmed the absence of difference in the rates of primary (*p* = 0.402) and secondary (*p* = 0.941) outcome.

**Conclusions:**

In patients with indications for dual-chamber pacemaker who require a high percentage of ventricular stimulation, RVA or non-RVA pacing resulted in comparable outcomes. This study is registered with ClinicalTrials.gov (identifier: NCT01647490).

## 1. Introduction

Chronic right ventricular apical (RVA) pacing is associated with an increased risk of atrial fibrillation, morbidity, and even mortality [1]. Several studies have shown that acute apical pacing in subjects with normal left ventricular systolic function results in mechanical dyssynchrony and decreased systolic function [2, 3].

Pacing at nonapical right ventricular (non-RVA) sites may yield more physiologic ventricular activation and less dyssynchrony and provide potential long-term benefits [4, 5]. Despite the lack of strong evidence, pacing at non-RVA sites has recently been adopted as standard procedure at many implanting centers [6]. However, major difficulties in placing the lead and accurately classifying [7] the final lead position have been described in the case of non-RVA pacing. Long-term outcomes are therefore unknown and potentially highly variable.

The aim of the Right Pace study was to compare the two approaches to chronic pacing of the right ventricle currently adopted in clinical practice: RVA and non-RVA pacing. In the present analysis, we compared the long-term outcome of right ventricular pacing according to the intended and actual lead position.

## 2. Methods

### 2.1. Study Design and Patients

The Right Pace study was a multicenter, prospective, single-blind, nonrandomized trial. A detailed description of the rationale and methods of the study has been published elsewhere [8]. Patients were included if they were indicated for dual-chamber pacemaker implantation according to the current guidelines and required a high percentage of ventricular pacing. Patients were excluded from the study if they had a previously implanted cardiac device, a conventional indication for an implantable defibrillator or cardiac resynchronization therapy, permanent atrial fibrillation, or a life expectancy lower than 2 years. The study was approved by the Institutional Review Boards of the participating centers and all subjects provided written consent.

Patients meeting eligibility requirements underwent baseline evaluation, which included demographics and medical history, clinical examination, and 12-lead electrocardiogram. They then underwent implantation of a dual-chamber pacemaker with standard right atrial and right ventricular leads. The target location for the right ventricular lead tip was the apex or the interventricular septum, according to the clinical practice of the center. Indeed, investigators were divided on the basis of their prior experience of nonapical pacing-lead implantation and the clinical practice adopted in their centers. X-rays in the anteroposterior, right oblique, and left oblique projections (>30°) were taken and stored. An echocardiographic evaluation was performed to assess the left ventricular dyssynchrony induced by right ventricular pacing. Dyssynchrony was detected by means of tissue Doppler imaging and measured as the delay between the septal and lateral peak systolic velocity curves (SLD). An SLD of 41 ms or more was defined as a significant left ventricular mechanical delay [9]. After discharge, clinic visits were scheduled every 6 months.

### 2.2. Endpoints

The primary endpoint was the composite of death due to any cause or hospitalization for heart failure. The secondary endpoint was the composite of death due to any cause, hospitalization for heart failure, or an increase in left ventricular end-systolic volume of 15% or more as compared with the baseline evaluation. Additional endpoints were the first episode of atrial fibrillation documented during the follow-up period and the ventricular pacing parameters.

### 2.3. Analysis of Final Pacing Lead Tip Position

X-rays and electrocardiograms were reviewed in independent core laboratories not directly involved in the implanting procedures. Lead position was adjudicated according to published criteria [7].

### 2.4. Statistical Analysis

All endpoints were initially analyzed as intention-to-treat, according to the lead site assigned at the implanting center. A predefined on-treatment analysis was also performed according to the final lead position, as determined by the lead adjudication committee. Descriptive statistics are reported as means ± SD for normally distributed continuous variables, or medians with 25th to 75th percentiles in the case of skewed distribution. Differences between mean data were compared by means of a *t*-test for Gaussian variables. The Mann-Whitney* U* test and the Wilcoxon nonparametric test were used to compare non-Gaussian variables for independent and paired samples, respectively. Differences in proportions were compared by applying chi-square analysis or Fisher's exact test, as appropriate. Hazard ratios (HRs) and their 95% confidence intervals (CIs) were computed by means of a Cox regression model, in which baseline predictors were considered as fixed covariates and the primary endpoints were considered as time-dependent covariates. We included in the multivariate Cox model any variable with *p* < 0.05 on univariate analysis. A *p* value < 0.05 was considered significant for all tests. All statistical analyses were performed by means of STATISTICA software, version 7.1 (StatSoft, Inc., Tulsa, OK, USA).

## 3. Results

### 3.1. Study Time-Lines and Population

From June 2012 through September 2014, 437 patients referred to the study centers for pacemaker implantation met eligibility requirements and underwent baseline evaluation. Of these, 274 were referred to centers routinely performing standard apical positioning of the right ventricular lead, while in 163 patients the target location for the right ventricular lead tip was the interventricular septum, in accordance with the clinical practice adopted in the remaining centers. Demographic data and clinical parameters were similar between the study groups at the time of enrollment (Table 1), except for a higher prevalence of diabetes in the non-RVA group. Moreover, the 2 study groups showed comparable levels of left ventricular dyssynchrony and QRS duration during spontaneous conduction and ventricular pacing.

### 3.2. Adjudication of the Lead Tip Position

X-rays were available for core laboratory adjudication of the lead position in 409 patients. Apical positioning of the right ventricular lead was not confirmed in 56 (20%) patients in the RVA group. Similarly, in 21 (16%) patients in the non-RVA group, the adjudicated pacing site was the apex. Therefore, the adjudicated RVA group numbered 239 patients and the adjudicated non-RVA group, 170 patients.

### 3.3. Effects on Primary and Secondary Endpoints

**Figure 1:**
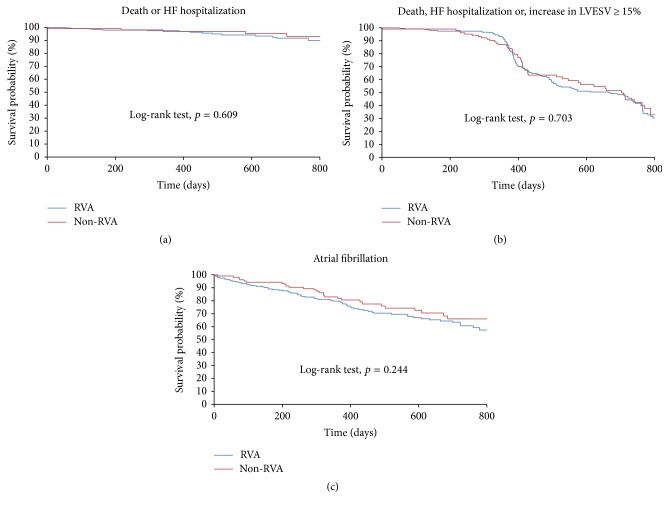
Kaplan-Meier estimate of time to death due to any cause or first hospitalization for heart failure (a), time to the composite of death due to any cause, hospitalization for heart failure, or a ≥15% increase in left ventricular end-systolic volume (b) and time to the first episode of documented atrial fibrillation (c).

Patients were followed up until December 2015. During a median follow-up of 19 months (25th to 75th percentiles, 13–25), 17 patients died (12 cardiac deaths and 5 noncardiac deaths). The rates of the primary outcome of death due to any cause or hospitalization for heart failure were comparable between groups (Figure 1; log-rank test, HR: 1.30; 95% CI: 0.50 to 3.38; *p* = 0.609), as were the rates of the composite of death due to any cause, hospitalization for heart failure, or an increase in left ventricular end-systolic volume of 15% or more as compared with the baseline evaluation (HR: 1.07; 95% CI: 0.77 to 1.48; *p* = 0.703). Comparison between the adjudicated RVA and adjudicated non-RVA groups confirmed the absence of difference in the rates of the primary (HR: 1.51; 95% CI: 0.60 to 3.76; *p* = 0.402) and secondary outcome (HR: 0.99; 95% CI: 0.72 to 1.35; *p* = 0.941). The pacing site, according to local or core laboratory assignment, did not show any association with outcomes on multivariate analysis (Table 2). Only the baseline ejection fraction turned out to be an independent determinant of the primary outcome (HR: 0.94, CI: 0.90 to 0.98; *p* = 0.002). Moreover, the risk of atrial fibrillation was comparable between groups. Figure 1 shows the Kaplan–Meier event-free curves regarding atrial fibrillation (log-rank test, HR: 1.30; 95% CI: 0.86 to 1.97; *p* = 0.244).

### 3.4. Pacing Data

No perioperative complication was reported by the investigators in both groups. During follow-up, 3 RV lead dislodgements were reported (1 in the RVA group and 2 in the non-RVA group). The pacing parameters were satisfactory in both groups at baseline and at the last observation, with pacing thresholds consistently below 1 V (Table 3). Nonetheless, the threshold was slightly lower in the Apex group.

## 4. Discussion

The present study showed that, in patients with indications for a dual-chamber pacemaker and requiring a high percentage of ventricular stimulation, pacing of the right ventricle at apical or nonapical right ventricular sites resulted in comparable clinical outcomes over 2 years.

The incidence of death, hospitalization for heart failure, or progression of heart failure, as measured by a significant increase in left ventricular end-systolic volume, was similar with standard RVA or alternative non-RVA positioning of the lead, in accordance with the clinical practice adopted in the centers.

Since the advent of intravenous leads, the apex has become the standard site for right ventricular lead implantation, as it ensures simple placement and greater lead stability and reliability. However, in subjects with normal left ventricular systolic function who require permanent pacing, RVA stimulation is associated with an increased risk of atrial fibrillation, morbidity, and even mortality [1]. These observations have raised questions regarding the appropriate pacing site.

Right ventricular septal placement has been proposed as an alternative approach for the safe implantation and possible easy extraction of pacemaker and implantable defibrillator leads [10, 11]. Moreover, a meta-analysis by Shimony et al. [4] suggested that non-RVA pacing might have beneficial effects on systolic function, though it also confirmed inconclusive results with respect to other outcome measures, such as exercise capacity, functional class, quality of life, and survival. More recently, Kaye et al. [12] compared traditional apical and septal pacing in patients with preserved baseline left ventricular function and found no significant lead position-related difference in systolic function over 2 years. Similarly, in patients who had undergone implantation of a defibrillator for cardiac resynchronization therapy, a non-RVA lead location yielded no benefit in terms of clinical outcome or echocardiographic response [13].

Despite the lack of strong evidence in favor of nonapical pacing and the difficulty of placing the lead and accurately classifying [7] the final lead position, pacing at non-RVA sites seems to have become a standard procedure at many implanting centers. According to a recent survey of the European Heart Rhythm Association, the apex was the first option for right ventricular lead positioning only in 47% of the participating centers [6]. This finding is in line with those of the European cardiac resynchronization therapy survey [14], which revealed that, also in the case of biventricular system implantation, the RVA position was adopted in no more than 74% of patients.

Therefore, the aim of the present study was to compare the effects of right ventricular pacing when the lead is positioned in the apex or at nonapical sites, according to the routine clinical practice of the participating centers. The investigators were divided on the basis of their prior experience of pacemaker implantation with apical or nonapical lead positioning, in order to reduce the bias related to the learning curve and to provide results applicable to routine clinical practice.

We demonstrated that the incidence of death, hospitalization for heart failure, or progression of heart failure over 2 years were similar with apical or non-RVA positioning of the lead. Therefore, we confirmed, in clinical practice, the negative results yielded by non-RVA pacing in the setting of a controlled clinical study [12]. We also confirmed the difficulty of placing the lead and classifying the final lead position, as agreement on pacing site classification between the implanting centers and subsequent central adjudication was only moderate. Nonetheless, we showed that the observed equivalence of RVA and non-RVA sites, in terms of outcome, could not be ascribed to inadequate lead positioning, as the analysis repeated after central reclassification of pacing sites confirmed the result.

The present findings extend our preliminary analysis of the acute echocardiographic effects of right ventricular pacing [15]. Indeed, we previously demonstrated that pacing of the RV at apical or nonapical sites resulted in increased intraventricular dyssynchrony of left ventricular contraction and that the degree of dyssynchrony induced was comparable between the pacing sites.

In the present study, about 50% of patients met the endpoint of heart failure progression at 2-year follow-up examination. A comparable rate of events was reported in patients with atrioventricular block, New York Heart Association (NYHA) class I, II, or III heart failure, and a left ventricular ejection fraction of 50% or less, enrolled in the Biventricular versus Right Ventricular Pacing in Heart Failure Patients with Atrioventricular Block (BLOCK HF) study [16]. However, in our population the baseline left ventricular ejection fraction was markedly higher than that reported in the BLOCK HF study (57% versus 40%). Therefore, the observed increase in left ventricular end-systolic volume during follow-up did not generally lead to severely depressed systolic dysfunction and thus was not paralleled by a high rate of death or hospitalization for heart failure.

The pacing site, according to local or core laboratory assignment did not show any association with outcomes on multivariate analysis. Only the baseline ejection fraction turned out to be an independent determinant of incipient heart failure. This suggests that patients requiring a high percentage of ventricular stimulation and with initial systolic dysfunction are at higher risk of further deterioration; non-RVA pacing seemed unable to prevent this worsening. Therefore, other pacing strategies should be investigated as alternative approaches to preventing the drawbacks of RVA stimulation.

Our results confirm previous findings [17, 18], in that we frequently observed new-onset atrial fibrillation in our pacemaker population. Nonetheless, we did not find any association between the incidence of atrial fibrillation and the ventricular pacing site.

The currently adopted approaches to ventricular pacing, RVA and non-RVA, seemed equally feasible. Indeed, we reported few lead-related adverse events, and the pacing parameters were satisfactory in both study groups at the baseline and at the last observation.

### 4.1. Study Limitations

The main limitation of the present study is the lack of randomization. However, the study was designed in order to obtain an unbiased representation of current clinical practice and to compare two approaches currently adopted as standard procedures at many implanting centers. Moreover, owing to the nonrandomized nature of the study, the groups were very different in size.

### 4.2. Conclusions

In patients with indications for a dual-chamber pacemaker who require a high percentage of ventricular stimulation, the two approaches currently adopted in clinical practice, that is, RVA and non-RVA pacing, resulted in comparable outcome and appeared equally safe.

## Figures and Tables

**Table 1:** Demographics and baseline characteristics of the study population.

Parameter	RVA (*n* = 274)	Non-RVA (*n* = 163)	*p*
Male gender, *n* (%)	171 (62)	104 (64)	0.770
Age, years	75 ± 9	73 ± 11	0.059
AV block—third degree, *n* (%)	45 (16)	39 (23)	0.054
AV block—second degree, *n* (%)	65 (24)	52 (32)	0.062
Coronary artery disease, *n* (%)	65 (24)	47 (29)	0.237
Myocardial infarction, *n* (%)	28 (10)	21 (13)	0.393
Previous valvular surgery, *n* (%)	12 (4)	5 (3)	0.493
History of atrial fibrillation, *n* (%)	66 (24)	33 (20)	0.353
Hypertension, *n* (%)	199 (73)	120 (74)	0.821
Diabetes, *n* (%)	65 (24)	53 (33)	0.045
Chronic obstructive pulmonary disease, *n* (%)	26 (9)	19 (12)	0.471
Chronic kidney disease, *n* (%)	56 (20)	29 (18)	0.499
LV ejection fraction, %	57 ± 9	58 ± 9	0.897
LVEDV, ml	101 ± 43	101 ± 30	0.992
LVESV, ml	44 ± 21	45 ± 18	0.695
Severe mitral regurgitation, *n* (%)	26 (9)	17 (10)	0.750
SLD > 41 ms during spontaneous conduction, *n* (%)	69 (25)	45 (28)	0.576
SLD > 41 ms during ventricular pacing, *n* (%)	132 (48)	83 (51)	0.579
QRS duration during spontaneous conduction, ms	98 ± 25	92 ± 24	0.122
QRS duration during ventricular pacing, ms	145 ± 30	141 ± 31	0.285
Active fixation lead in right ventricle, *n* (%)	74 (27)	135 (83)	<0.001

AV = atrioventricular; LV = left ventricular; LVEDV = left ventricular end-diastolic volume; LVESV = left ventricular end-systolic volume; SLD = septal-to-lateral delay.

**Table 2:** Univariate and multivariate analysis of factors associated with death due to any cause or hospitalization for heart failure.

	Univariate analysis			Multivariate analysis		
	HR	95% CI	*p*	HR	95% CI	*p*
Male gender	0.70	0.29–1.67	0.421	-	-	-
Age	1.06	1.00–1.13	0.049	1.06	1.00–1.12	0.051
Coronary artery disease	1.55	0.61–3.93	0.355	-	-	-
History of atrial fibrillation	0.32	0.08–1.37	0.128	-	-	-
Hypertension	1.81	0.53–6.15	0.342	-	-	-
Diabetes	1.30	0.51–3.29	0.582	-	-	-
Chronic obstructive pulmonary disease	1.45	0.43–4.94	0.550	-	-	-
Chronic kidney disease	1.03	0.34–3.10	0.952	-	-	-
LV ejection fraction	0.94	0.90–0.97	0.001	0.94	0.90–0.97	0.002
Severe mitral regurgitation	1.04	0.24–4.45	0.961	-	-	-
Spontaneous SLD > 41 ms	0.79	0.25–2.48	0.687	-	-	-
Paced SLD > 41 ms	1.36	0.50–3.66	0.546	-	-	-
Spontaneous QRS duration	1.00	0.99–1.02	0.529	-	-	-
Paced QRS duration	1.01	0.99–1.02	0.440	-	-	-
Ventricular pacing percentage	1.00	0.99–1.01	0.200	-	-	-
Nonapical pacing	1.30	0.48–3.56	0.610	-	-	-
Adjudicated nonapical pacing	1.51	0.58–3.95	0.405	-	-	-

LV = left ventricular; SLD = septal-to-lateral delay.

**Table 3:** Pacing parameters.

Parameter	Baseline			Last follow-up exam		
	RVA (*n* = 274)	Non-RVA (*n* = 163)	*p*	RVA (*n* = 274)	Non-RVA (*n* = 163)	*p*
Sensed R wave amplitude, mV	12 ± 5	11 ± 5	0.170	11 ± 6	10 ± 5	0.372
RV lead impedance, Ohm	725 ± 269	635 ± 177	<0.001	511 ± 129	538 ± 178	0.182
RV pacing threshold amplitude, V	0.5 ± 0.4	0.6 ± 0.4	<0.001	0.7 ± 0.6	0.9 ± 0.5	0.010
RV pacing threshold duration, ms	0.4 ± 0.1	0.5 ± 0.1	<0.001	0.4 ± 0.2	0.5 ± 0.2	0.216

RV = right ventricular.

## Appendix

Enrolling centers and investigators are as follows:

  Clinica Città di Alessandria, Alessandria: Paolo Diotallevi, Enrico Gostoli;
  Cliniche Humanitas Gavazzeni, Bergamo: Giosuè Mascioli;
  Ospedale Maggiore, Bologna: Valeria Carinci, Francesco Pergolini;
  Ospedale SS. Trinità, Borgomanero (NO): Paola Paffoni, Umberto Parravicini;
  Ospedale Poliambulanza, Brescia: Domenico Pecora;
  A.O.U.P. “Vittorio Emanuele” - Ospedale Ferrarotto, Catania: Valeria Calvi, Angelo Di Grazia, Claudio Liotta, Francesca Scarfia, Giuseppe D'Angelo;
  A.O. Cannizzaro, Catania: Francesco Lisi, Francesco Liberti;
  Ospedale Pugliese-Ciaccio, Catanzaro: Giampiero Maglia, Vittorio Aspromonte, Francesco Arabbia;
  Fondazione Istituto San Raffaele-G. Giglio, Cefalù (PA): Riccardo Airò Farulla, Gabriele Giannola;
  Ospedale Bufalini, Cesena (FC): Paolo Sabbatani;
  Ospedale San Paolo, Civitavecchia (RM): Sergio Calcagno, Giovanni Biscotti;
  Ospedale S. Anna, Como: Giovanni Luca Botto, Giovanni Russo, Barbara Mariconti;
  Ospedale Maggiore, Crema (CR): Giuseppe Inama, Antonio Foffa;
  Ospedale di Desenzano del Garda, Desenzano del Garda (BS): Gian Paolo Gelmini, Tommaso Bignotti;
  Ospedale Civile “La Memoria”, Gavardo (BS): Marco Racheli, Loretta Brentana;
  Ospedale Civile di Gorizia, Gorizia: Luca Perazza;
  Ospedale Versilia, Lido di Camaiore (LU): Alessio Lilli, Marco Tullio Baratto;
  Ospedale Carlo Poma, Mantova: Albino Reggiani;
  Ospedali Riuniti Papardo, Messina: Santina Patanè;
  Ospedale S. Maria di Loreto Mare, Napoli: Bernardino Tucillo, Raimondo Calvanese, Michelangelo Canciello, Maria Accadia, Raffaele Iengo;
  Seconda Università degli Studi di Napoli - Ospedale Monaldi, Napoli: Gerardo Nigro, Vincenzo Russo, Anna Rago;
  Ospedale Sacro Cuore, Negrar (VR): Alessandro Costa, Giulio Molon;
  Ospedale S. Maria della Pietà, Nola (NA): Carmine Muto, Vincenzo Ruggiero, Luigi Caliendo, Francesco Scafuro;
  Ospedale Civile San Martino, Oristano: Francesco Dettori, Antonio Caddeo, Gianfranco Delogu;
  A.O.U.P. “Paolo Giaccone” - Università degli Studi di Palermo, Palermo: Gianfranco Ciaramitaro, Giuseppe Coppola, Stefano Augugliaro, Dario Orlando;
  ARNAS Ospedale Civico, Palermo: Umberto Giordano, Giuseppe Sgarito;
  Ospedale San Pietro Fatebenefratelli, Roma: Lorenzo Maria Zuccaro, Daniele Porcelli;
  A.O. Santa Maria, Terni: Giovanni Carreras, Chiara Marini;
  Ospedale Maria Vittoria, Torino: Massimo Giammaria, Maria Teresa Lucciola.
